# Biological cost of aminoglycoside resistance Arm/Kam 16S rRNA methyltransferases from natural antibiotic producers and clinical pathogens

**DOI:** 10.1128/aac.00742-25

**Published:** 2025-07-31

**Authors:** Darija Vidučić, Sonja Obranić, Mihaela Matovina, Fedora Babić, Gordana Maravić-Vlahoviček

**Affiliations:** 1 Department of Biochemistry and Molecular Biology, Faculty of Pharmacy and Biochemistry, University of Zagreb 37631, Zagreb, Croatia; 2 University Centre Varaždin, University North 325073 , Varaždin, Croatia; 3 Division of Organic Chemistry and Biochemistry, Ruđer Bošković Institute 54583, Zagreb, Croatia; Columbia University Irving Medical Center, New York, New York, USA

**Keywords:** bacterial fitness, fitness cost, aminoglycoside resistance, 16S rRNA methyltransferases

## Abstract

16S rRNA methyltransferases have emerged as critical elements of high-level aminoglycoside resistance in clinical pathogens. We investigated the fitness costs associated with the expression of six methyltransferases isolated from clinical strains (ArmA, RmtA, RmtB, RmtC, RmtD, and NpmA), and two methyltransferases from natural antibiotic producers (Sgm and KamB) in *Escherichia coli*. Growth competition assays revealed that methyltransferases found in natural producers imposed significantly lower fitness costs than those isolated from clinical strains, allowing resistant populations to persist at stable levels. Translational fidelity assays demonstrated that most methyltransferases induce error-prone phenotypes by allowing increased readthrough of nonsense codons and frameshift mutations, while KamB uniquely increased translational accuracy. Deletion of the housekeeping methyltransferase RsmF further altered these effects, highlighting the complex interplay between endogenous and exogenous methylation processes. Stress response experiments showed varying results: most methyltransferases increased susceptibility to hyperosmotic stress, while several (RmtB, RmtA, ArmA, and KamB) increased tolerance to acidic stress. These findings reveal that 16S rRNA methyltransferases play complex roles in bacterial physiology beyond antibiotic resistance, with important implications for the persistence of resistance and potential therapeutic strategies targeting specific vulnerabilities in resistant bacteria.

## INTRODUCTION

The extensive and often indiscriminate use of antibiotics in human medicine, along with their widespread application in agriculture and veterinary medicine, has contributed to the development and global spread of antibiotic-resistant pathogenic bacterial strains (1, 2). Aminoglycosides are a widely used class of antibiotics that are effective against infections caused by gram-negative and gram-positive bacteria. Because of their important function in the treatment of severe bacterial infections, aminoglycosides have been classified by the World Health Organization as critically important antimicrobials for human medicine (3).

Bacterial resistance to aminoglycosides occurs through multiple mechanisms, with enzymatic inactivation by aminoglycoside-modifying enzymes (AMEs) being the most prevalent. Other resistance strategies include reduced membrane permeability, antibiotic efflux, and ribosomal target modification by 16S rRNA methyltransferases—the most recently identified mechanism in clinical settings. While AMEs are the most common resistance mechanism, 16S rRNA methyltransferases play a critical role in conferring high-level aminoglycoside resistance in clinical environments (4, 5). These methyltransferases modify specific nucleotides within helix 44 of the 16S rRNA and are divided into two families: the Arm family, which methylates nucleotide G1405, and the Kam family, which methylates nucleotide A1408.

In clinical settings, 16S rRNA methyltransferases were first reported in 2003, when two enzymes, ArmA and RmtA, were identified in multidrug-resistant *Klebsiella pneumoniae* and *Pseudomonas aeruginosa* strains, respectively (6, 7). Since then, additional enzymes from both families have been identified, including members such as ArmA, RmtA-RmtI (Arm family), and NpmA, NpmB1/2, NpmC and WarA (Kam family), all of which play a significant role in conferring high-level resistance to aminoglycosides in clinical settings (7–18). However, these enzymes are not limited to clinical environments. They are also naturally produced by aminoglycoside-producing actinomycetes, such as those from the genera *Streptomyces* and *Micromonospora*, which use them to protect their ribosomes from the toxic effects of their own antibiotic products by methylating nucleotides within the aminoglycoside binding site of helix 44 in the 16S rRNA (19–24). In antibiotic-producing bacteria, such as *Streptomyces*, resistance enzymes often have a natural role in self-protection, a concept first proposed by Julian Davies and Raoul Benveniste in 1973, suggesting that such mechanisms in producers could be the ancestral origins of resistance in clinical pathogens (25).

Bacteria can become antibiotic-resistant through adaptive evolution, an incremental genetic alteration over a sequence of generations caused by the exposure to an antibiotic, or by horizontal gene transfer, where resistance-conferring genes are passed between various bacterial species (26). The mechanisms of bacterial resistance to antibiotics are often associated with a metabolic burden, which refers to the energy costs that bacteria must tolerate to maintain their resistance. This burden can cause a decrease in bacterial fitness, which is an organism’s ability to survive and grow in a given environment (27). Antibiotics provide a selective advantage to resistant bacteria; however, in their absence in the environment, their fitness can be decreased, making them disadvantaged (28). The resistance dynamics are influenced by the conditions of the environment. In environments where there are no antibiotics, decreased fitness may result in progressive loss of resistance among the bacterial population. However, if the metabolic burden reduces over time, resistant bacteria will be able to survive in the absence of antibiotics, thereby making it easier to spread antimicrobial resistance (29). The spread of resistance is further facilitated by the occurrence of antibiotics in some environments through contamination, which can impose ongoing selective pressure for resistance maintenance even outside clinical environments (30).

**Fig 1:**
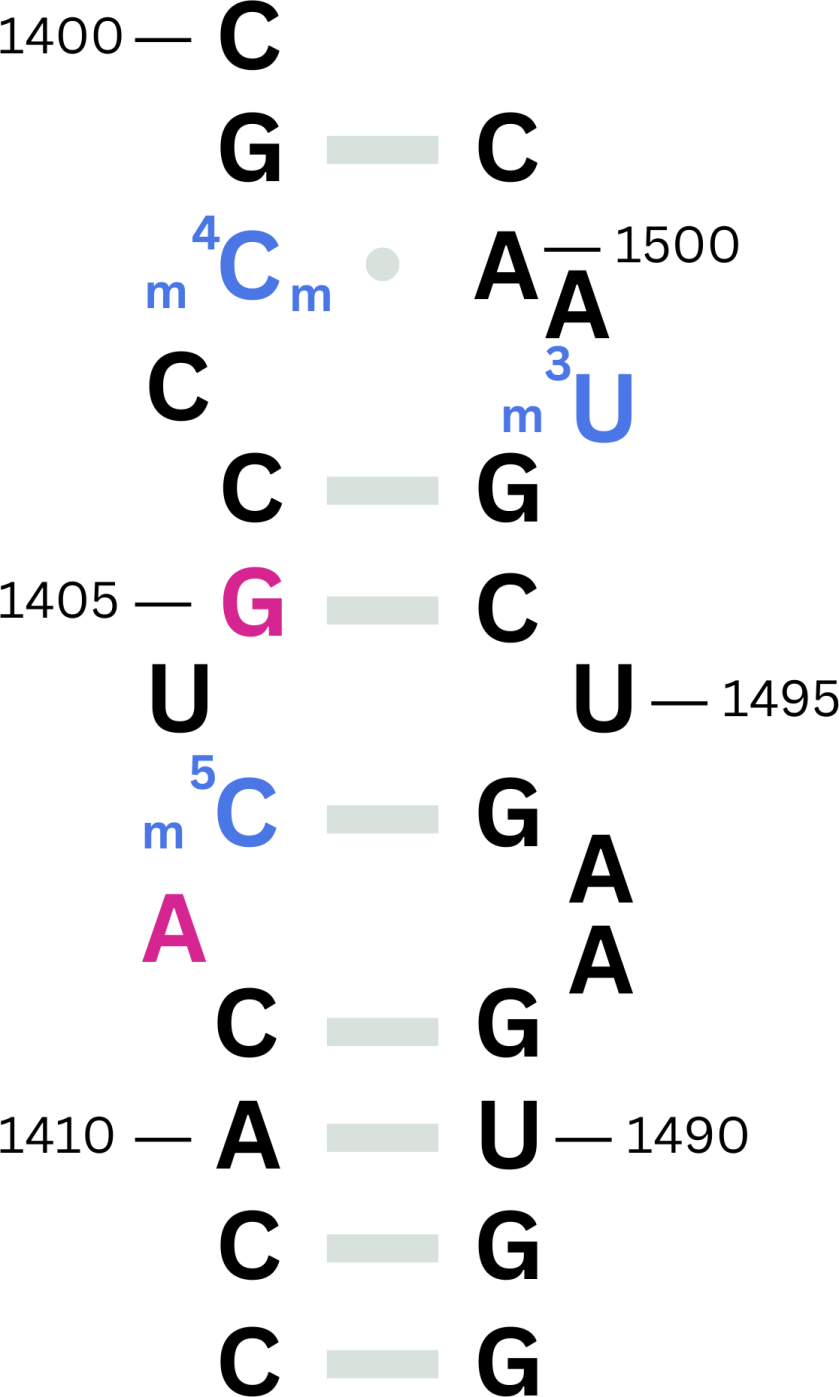
Segment of helix 44 in the A site of *E. coli* 16S rRNA. Nucleotides methylated by housekeeping methyltransferases are highlighted in blue (C1402, C1407, and U1498), while nucleotides methylated by aminoglycoside resistance rRNA methyltransferases are shown in magenta (G1405 and A1408). This region forms the aminoglycoside binding pocket and is a critical target for both endogenous rRNA modification and acquired antibiotic resistance mechanisms.

Bacterial rRNA harbors several nucleotide modifications that are critical to the functional dynamics of the ribosomes, including facilitating ribosomal subunit assembly, resistance to antimicrobial agents, and ensuring correct structural conformation of functional domains (31). In the vicinity of nucleotides A1408 and G1405 in the 16S rRNA are four nucleotides targeted by the housekeeping methyltransferases RsmF (C1407), RsmI and RsmH (C1402), and RsmE (U1498) (32). Figure 1 shows a part of helix 44 in the A site of 16S rRNA, highlighting the region in the vicinity of nucleotides G1405 and A1408, which are critical for aminoglycoside antibiotic binding. Several studies have shown that resistance methyltransferases of the Arm and Kam families interfere with the activities of the housekeeping methyltransferases and block their common functions (33–36). Therefore, it would be reasonable to suggest that the competition between resistance and housekeeping methyltransferases significantly influences bacterial fitness and resistance profiles, highlighting the intricate balance between antibiotic resistance mechanisms and optimal ribosomal function in bacterial populations.

Although the clinical significance of 16S rRNA methyltransferases in conferring high-level resistance to aminoglycosides has been well established, the relative fitness costs of various methyltransferases remain incompletely characterized. Previous research has addressed single methyltransferases, yielding results that are occasionally contradictory regarding their impact on bacterial fitness (33, 35–37). The observed variability in fitness costs associated with the expression of various 16S rRNA methyltransferases motivated us to conduct a detailed analysis of their effects on bacterial physiology and viability. In this study, we investigated the impact of different methyltransferases (RmtA, RmtB, RmtC, RmtD, Sgm, KamB, NpmA, and ArmA) on *Escherichia coli* grown under non-selective conditions. In addition, we investigated these effects in *E. coli* cells lacking the housekeeping methyltransferase RsmF, to determine the extent to which the absence of this endogenous modification affects resistance levels and fitness costs, and to specifically evaluate the impact of Arm and Kam enzymes on these cells. Our research focused on how these enzymes affect bacterial growth, translational fidelity, and stress responses. By clarifying the complex trade-offs between antibiotic resistance and bacterial fitness, we aimed to gain insight into the evolutionary paths of resistance genes and their consequences for public health, especially given the rise of antimicrobial resistance. This work will advance our understanding of how various methyltransferases influence bacterial survival and adaptation in different environments, and thus ultimately guide strategies to counter antibiotic resistance.

## RESULTS

### Growth rates and growth competition assay

**Fig 2:**
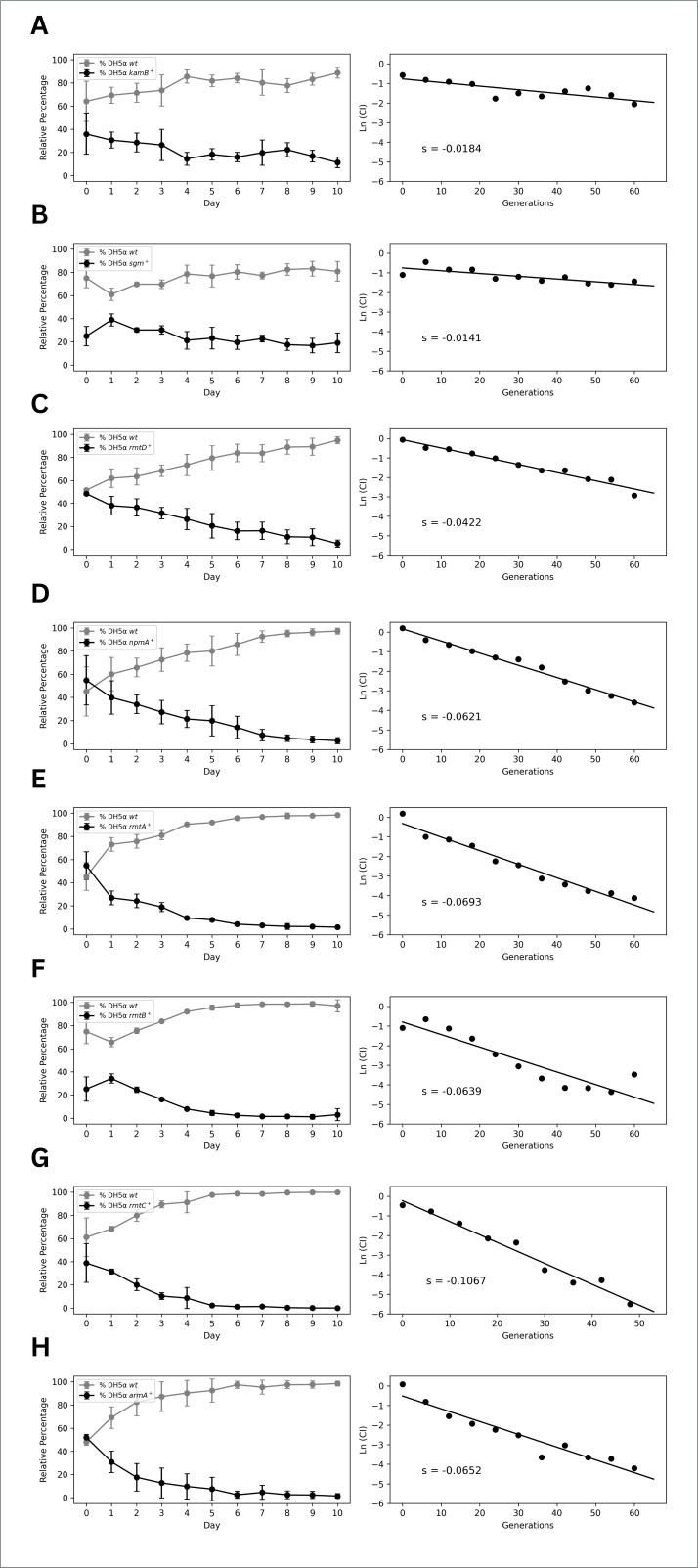
Growth competition assays and fitness cost of 16S rRNA methyltransferases expressed in *E. coli*. Left panels show the relative percentages of wild-type strains (gray) versus strains expressing methyltransferases (black) over 10 days of competition. Error bars represent standard deviations. Right panels show the natural logarithm of the competitive index (Ln CI) plotted against generations, with the slope (S) indicating the magnitude of fitness cost. Panels (A–H) represent different methyltransferases: (A) (KamB) and (B) (Sgm) show the lowest fitness costs, (C) (RmtD) and (D) (NpmA) show intermediate fitness costs, and (E) (RmtA), (F) (RmtB), (G) (RmtC), and (H) (ArmA) show the highest fitness costs.

Growth rates were measured during the exponential phase for cells carrying either an empty plasmid or a plasmid encoding a 16S rRNA methyltransferase and were expressed as relative values compared to the parental strain. A two-way ANOVA of the monoculture growth times showed a statistically significant interaction between the categories of *E. coli* strain and the type of plasmid (*F* = 3.04, *P* = 0.0014), as well as a significant main effect of the expressed methyltransferase (*F* = 2.57, *P* = 0.020), but no significant main effect of *E. coli* strain alone (*F* = 1.88, *P* = 0.16). Post hoc comparisons (Dunnett tests), however, did not reveal statistically significant differences between individual groups. In contrast, growth competition assays revealed variable fitness costs for the eight 16S rRNA methyltransferases tested (ArmA, RmtA, RmtB, RmtC, RmtD, Sgm, KamB, and NpmA) when expressed in *E. coli* cells. For all the methyltransferases, when the strains carrying the empty-plasmid controls were considered, they competed better than the strains that expressed the methyltransferases. However, the degree and rate of such competitive advantages varied for each of the methyltransferases tested under the non-selective conditions of all the experiments. The results of the growth rate experiments are presented in Table 1, and the results of the growth competition assay are shown in Fig. 2. In these assays, the competitive index (ln(CI)) for each strain was plotted against the number of generations, and the slope (s) of this relationship provides a quantitative measure of the fitness cost associated with each methyltransferase. A more negative slope indicates a greater reduction in competitive fitness over time.

**TABLE 1:** Relative growth rates of *E. coli* DH5α, BW25113, and BW25113/*ΔrsmF* strains expressing various 16S rRNA methyltransferases^*a*^

Strain	Relative growth rate
DH5α	1.00
DH5α/*sgm*	1.05 ± 0.01
DH5α/*rmtA*	1.06 ± 0.01
DH5α/*rmtB*	0.99 ± 0.05
DH5α/*rmtC*	0.96 ± 0.02
DH5α/*rmtD*	1.06 ± 0.03
DH5α/*armA*	0.96 ± 0.03
DH5α/*kamB*	0.93 ± 0.03
DH5α/*npmA*	0.94 ± 0.04
BW25113	1.00
BW25113/*sgm*	1.03 ± 0.06
BW25113/*rmtA*	1.01 ± 0.03
BW25113/*rmtB*	0.96 ± 0.04
BW25113/*rmtC*	0.93 ± 0.03
BW25113/*rmtD*	0.97 ± 0.05
BW25113/*armA*	0.98 ± 0.01
BW25113/*kamB*	1.05 ± 0.01
BW25113/*npmA*	0.99 ± 0.08
BW25113 ∆*rsmF*	1.00
BW25113 ∆*rsmF*/*sgm*	0.95 ± 0.02
BW25113 ∆*rsmF*/*rmtA*	1.00 ± 0.01
BW25113 ∆*rsmF*/*rmtB*	0.97 ± 0.05
BW25113 ∆*rsmF*/*rmtC*	1.02 ± 0.06
BW25113 ∆*rsmF*/*rmtD*	0.96 ± 0.02
BW25113 ∆*rsmF*/*armA*	1.01 ± 0.03
BW25113 ∆*rsmF*/*kamB*	0.95 ± 0.02
BW25113 ∆*rsmF*/*npmA*	0.90 ± 0.02

^*a*^ Growth rates were measured during the exponential phase and are expressed as values relative to the corresponding parental strain (set to 1.00). Data represent mean ± standard deviation from at least three independent experiments.

Table 1 summarizes comparative growth rate values for *E. coli* DH5α and BW25113 cells as well as *E. coli* BW25113 cells that lack RsmF methyltransferase (*ΔrsmF*), that express various 16S rRNA methyltransferases. In the genetic background of DH5α, expression of most methyltransferases yielded a slight reduction or no significant change from the parental strain growth rate, with the most notable decreases observed in DH5α/*kamB*, DH5α/*npmA*, and DH5α/*rmtC* (0.93 ± 0.03, 0.94 ± 0.04, and 0.96 ± 0.02, respectively, compared to wild-type at 1.00). In contrast, expression of Sgm, RmtA, and RmtD methyltransferases resulted in growth rates slightly above wild-type, with notable increases observed for RmtA and RmtD (both 1.06 ± 0.01 and 1.06 ± 0.03, respectively). In the genetic background of BW25113, expression of most methyltransferases yielded a slight reduction or no significant change from the parental strain growth rate, with the most notable decreases observed in BW25113/*rmtC*, BW25113/*rmtB*, and BW25113/*rmtD* (0.93 ± 0.03, 0.96 ± 0.04, and 0.97 ± 0.05, respectively, compared to wild-type at 1.00). In contrast, expression of Sgm, RmtA, and KamB methyltransferases resulted in growth rates closer to or slightly above wild-type, with a notable increase observed for KamB (1.05 ± 0.01). In the background of BW25113 *ΔrsmF*, relative growth rate values were primarily similar to or slightly below the parental strain, with BW25113 *ΔrsmF*/*rmtC* and BW25113 *ΔrsmF*/*armA* showing slight increases (1.02 ± 0.06 and 1.01 ± 0.03, respectively), while BW25113 *ΔrsmF*/*npmA*, BW25113 *ΔrsmF*/*sgm*, and BW25113 *ΔrsmF*/*kamB* showed the most notable reductions in the growth rates (0.90 ± 0.02, 0.95 ± 0.02, and 0.95 ± 0.02, respectively).

The most severe fitness costs were imposed by methyltransferases RmtA, RmtB, RmtC, NpmA, and ArmA, as they were associated with a fast decline of the resistant strains. For RmtC methyltransferase, the proportion of resistant strain was initially 38.86% on Day 0, which decreased to 8.62% on Day 4 and then stabilized at a low proportion of around 1% from Day 6 onward. The same trend was observed for the methyltransferase RmtB; it was initially 25.19%, decreased to 7.95% by Day 4, and further decreased to 3.02% by Day 10. The strain carrying NpmA was initially at a higher proportion of 54.79%, but it also displayed the same trend of decline as observed with RmtA and RmtB. Its ratio decreased to 21.37% on Day 4 and reached 2.66% by Day 10. ArmA methyltransferase also had a very close pattern to that of RmtA and NpmA. It began at 51.95% and decreased to 17.58% on Day 2 and 9.67% on Day 4. On Day 10, the proportion of strains expressing ArmA was merely 1.47%, whereas the control strain was predominant, constituting approximately 98.53% of the strains. The results indicate that expressing RmtA, RmtB, RmtC, NpmA, and ArmA methyltransferases was tremendously costly to bacterial cells in terms of bacterial fitness. The energy costs of plasmid expression and maintenance, as well as the potential interference of methyltransferase activity with ribosomal function, are likely to be the primary causes of these high levels of fitness costs.

The proportion of the strain harboring RmtD methyltransferase fell from 48.45% on Day 0 to 26.51% on Day 4, eventually settling at 5.05% on Day 10. Such an intermediate rate of decline in population indicates that RmtD methyltransferase imposed a moderate fitness cost compared to RmtA, RmtB, RmtC, NpmA, and ArmA methyltransferases.

Sgm and KamB methyltransferases isolated from natural antibiotic producers both present a comparatively low fitness cost, enabling their resistant strains to be continuously detectable throughout the duration of the experiment. The strain that expressed the Sgm methyltransferase reached stabilization at 19.19% by Day 10 and hence presented the lowest fitness cost of all of the studied methyltransferases. Stabilization of KamB by Day 10 at 11.37% is indicative of a moderate fitness cost for strains that produce this enzyme. Presumably, both Sgm and KamB have fewer metabolic and ribosomal effects than the other tested methyltransferases, allowing small but stable populations of resistant bacteria in mixed cultures.

### Aminoglycoside resistance levels

To determine the functional activity of the 16S rRNA methyltransferases as a proxy for 16S rRNA target site modification, minimum inhibitory concentrations (MICs) for kanamycin, as a representative aminoglycoside, were determined against *E. coli* strains that expressed each methyltransferase. In our previous work, we reported the kanamycin MICs for Sgm, NpmA, KamB, RmtB, RmtC, RmtD, and ArmA methyltransferases (38, 39) using the pBBR1MCS-3_START plasmid (data for RmtA was collected, but not published). During that study, we also determined kanamycin MICs for methyltransferase genes expressed in the pBBR1MCS-4_START plasmid, which we present here for the first time. The results from both vector systems are consistent, confirming the reliability of our findings across these closely related plasmids. Table 2 displays a recap of MIC values of kanamycin with high-level resistance relative to the control strain as anticipated for activity of 16S rRNA methyltransferases.

**TABLE 2:** Minimum inhibitory concentrations (MICs) of kanamycin for *Escherichia coli* strains expressing 16S rRNA methyltransferases

Methyltransferase	Kanamycin MIC (mg/L)
Control (empty plasmid)	2
Sgm	>1,024
RmtA	>1,024
RmtB	>1,024
RmtC	>1,024
RmtD	>1,024
ArmA	>1,024
KamB	512
NpmA	>1,024

### Translational fidelity

Translational accuracy was quantitatively assessed in isogenic *E. coli* DH5α strains and a Δ*rsmF* mutant lacking the housekeeping 16S rRNA methyltransferase, by employing β‐galactosidase reporter assays. In this study, each aminoglycoside resistance methyltransferase—Sgm, RmtA, RmtB, RmtC, RmtD, ArmA, NpmA, and KamB—was expressed from a stable, broad-range plasmid pBBR1MCS-4_START (38). Reporter plasmids carrying modified *lacZ* alleles were used: pSG 3/4 had a UGA stop codon, pSG 413 had a CUG codon mutation, and pSG 12DP had a −1 frameshift mutation, thereby allowing the quantitation of the efficiency of ribosomal readthrough events as relative Miller units compared to control strains that carried empty plasmids.

**Fig 3:**
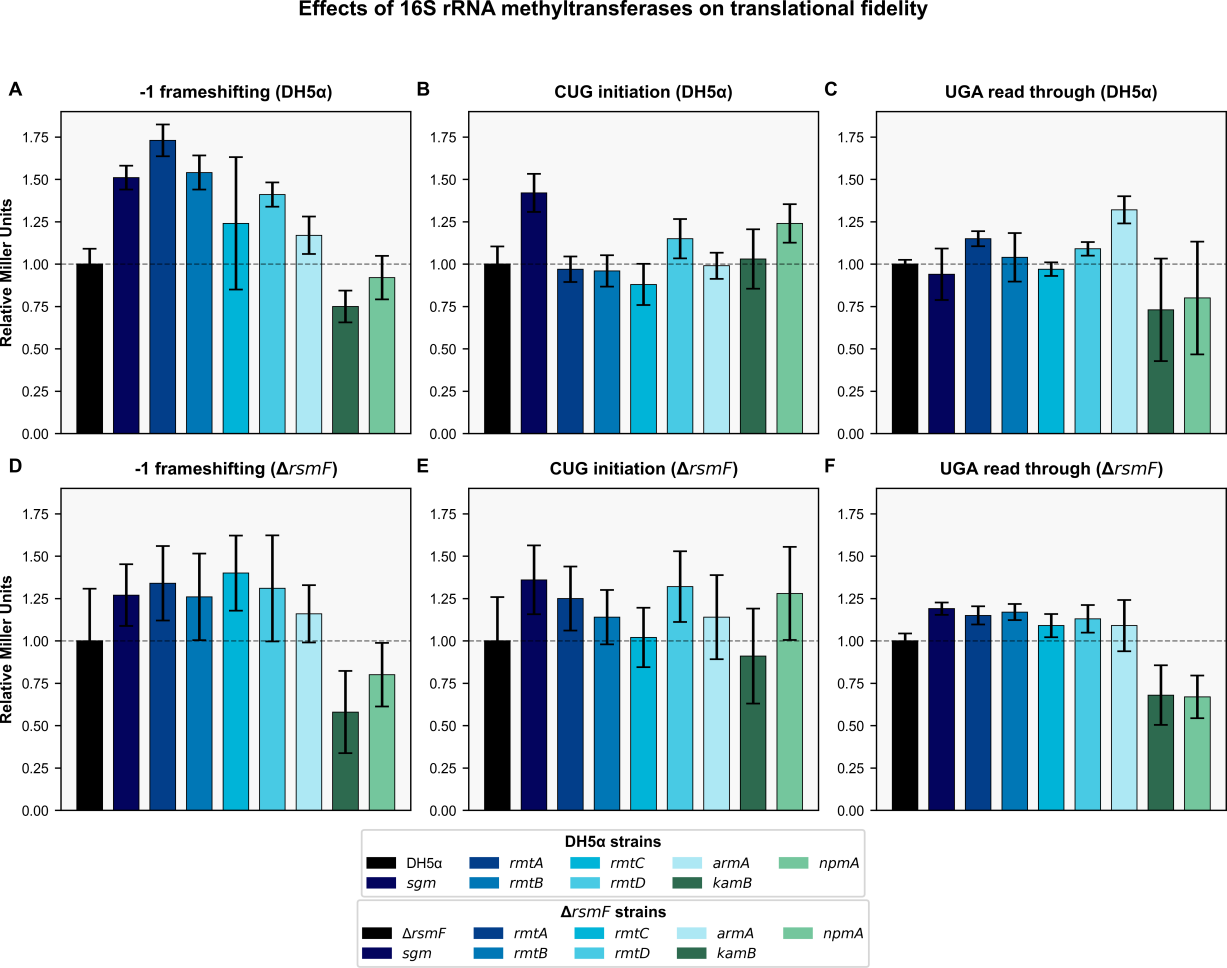
Effects of 16S rRNA methyltransferases on translational fidelity in *E. coli*. Bar charts showing the relative β-galactosidase activity (in Miller units) for different translational accuracy parameters in DH5α (**A through C**) and *ΔrsmF* (**D through F**) strains expressing various 16S rRNA methyltransferases. (**A and D**) −1 frameshifting efficiency; (**B and E**) CUG codon readthrough; (**C and F**) UGA stop codon readthrough. Values are normalized to control strains (DH5α or *ΔrsmF* without methyltransferase expression) set to 1.0, indicated by dashed lines. Error bars represent the standard deviation (*n* = 3). Gene names for methyltransferases are shown in italics in the legend.

In wild-type DH5α cells, the baseline β-galactosidase activity from a wild-type *lacZ* reporter (pSG 25) was stable regardless of methyltransferase expression, thus ensuring that the overall process of translation was not being impaired by plasmid maintenance. However, the expression of specific methyltransferases significantly affected the frequency of translational errors. For instance, expression of ArmA increased UGA readthrough by 32% relative to the control. Sgm expression promoted CUG initiation significantly by 42%, whereas RmtA and RmtB were both associated with marked increase in −1 frameshift, with RmtA displaying a 72% increase and RmtB displaying a 55% increase; Sgm also contributed a 52% rise in frameshift events. Our results suggest that, under wild-type conditions, the methyltransferases Sgm, RmtA, RmtB, ArmA, and to a lesser degree RmtC and NpmA, push the translation apparatus into an error-prone state. Figure 3 presents the effects of various 16S rRNA methyltransferases on translational fidelity in *E. coli*.

When these methyltransferases were expressed in the *ΔrsmF* background, the error profile of translation was altered in a different manner. Whereas RmtA, Sgm, and RmtB still increased UGA readthrough (by 15–18%), the absence of *rsmF* reduced UGA readthrough for ArmA by 23%, suggesting interaction between the endogenous and exogenous methyltransferase activities. In addition, the lack of *rsmF* increased Sgm’s effect on CUG initiation to give a 35% increase, whereas NpmA expression in this mutant background resulted in a 28% increase in CUG initiation. Notably, RmtC expression in *ΔrsmF* cells resulted in a substantial 40% increase in −1 frameshift. In contrast to the other enzymes, expression of KamB caused reduced −1 frameshift efficiency by 22% in the wild-type strain and by 40% in the *ΔrsmF* mutant, which is indicative of an error-restrictive phenotype.

### Stress response

In order to analyze the impact of 16S rRNA methyltransferases on the bacterial stress response, we subjected *E. coli* DH5α strains expressing various methyltransferases (specifically RmtA, RmtB, RmtC, RmtD, ArmA, Sgm, NpmA, and KamB) to hyperosmotic and acid stress. These conditions mimic the external environmental stresses and physiological challenges encountered in the human gastrointestinal tract, such as high acidity in the upper intestine and high osmolarity in the colon.

**Fig 4:**
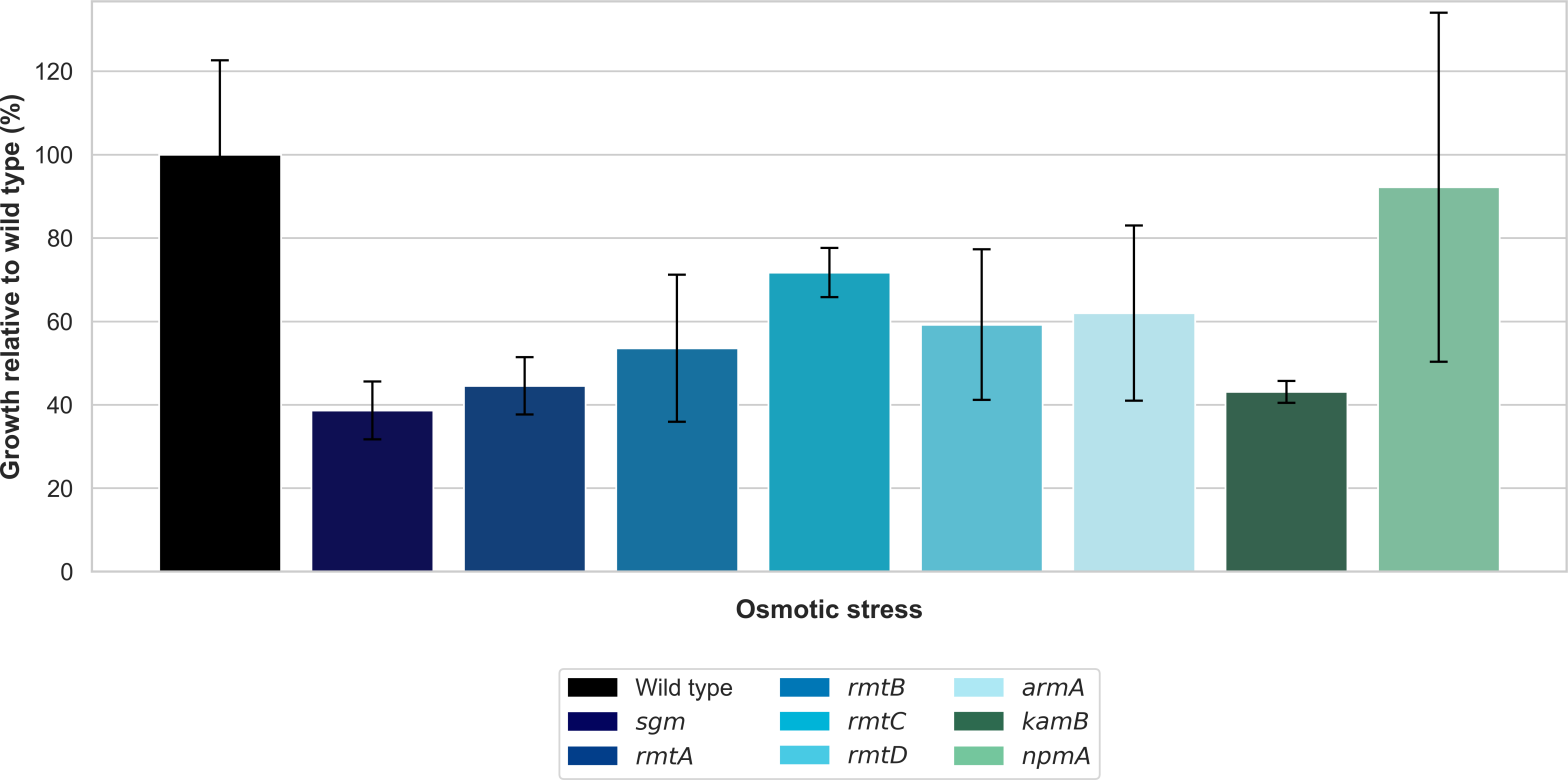
Growth of *E. coli* DH5α strains expressing different methyltransferases after exposure to hyperosmotic conditions (300 mM NaCl) for 3 h. Values represent the mean percentage of growth after stress, normalized to the wild-type strain (set to 100%), with error bars indicating standard deviation.

Our results showed drastic variation in the tolerance to hyperosmotic stress between strains (Fig. 4). While wild-type cells grew well in hyperosmotic conditions (100% relative to *t* = 0), most methyltransferase-expressing strains were hypersensitive to hyperosmotic conditions. Strains expressing Sgm, RmtA, and KamB had severely inhibited growth (38.6%, 44.5%, and 43.1%, respectively), while RmtB, RmtD, and ArmA exhibited moderately inhibited growth (53.6%, 59.2%, and 62.0%, respectively) relative to wild-type cells. The RmtC-producing strain had a less pronounced effect (71.7%), whereas, interestingly, the NpmA-expressing strain exhibited growth comparable to wild-type cells (92.2%), suggesting this particular methyltransferase does not compromise osmotic stress tolerance.

**Fig 5:**
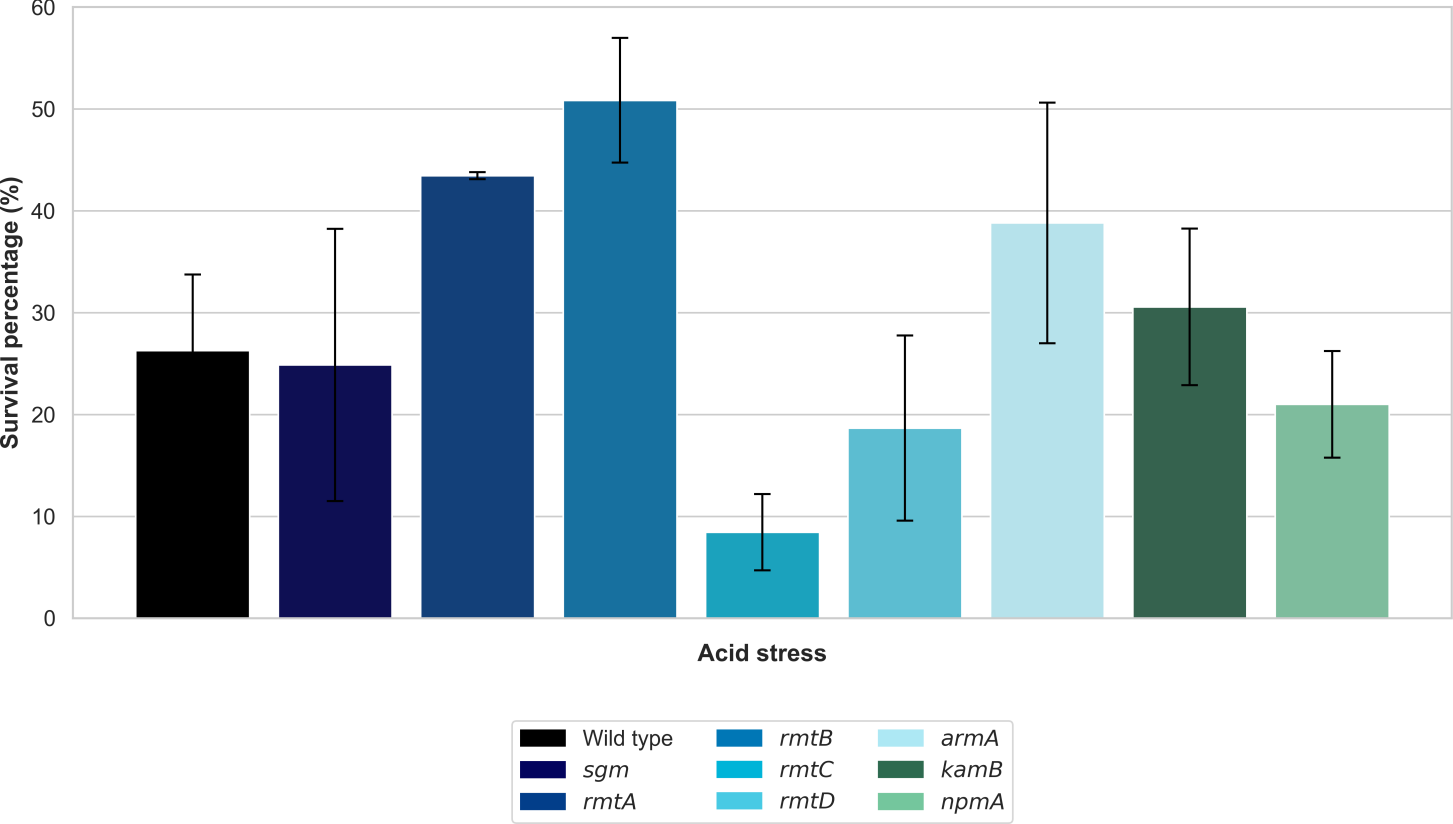
Effect of 16S rRNA methyltransferases on acid stress tolerance in *E. coli*. Survival percentages of *E. coli* DH5α strains expressing different methyltransferases after exposure to acid stress (pH 3.0) for 1 h. Values represent the mean percentage of surviving cells relative to pre-stress conditions (*t* = 0, set to 100%), with error bars indicating standard deviation.

In contrast to hyperosmotic stress, the acid stress experiments revealed that several methyltransferases were associated with enhanced acid tolerance (Fig. 5). Wild-type cells exhibited a survival rate of merely 26.3% under acid shock, whereas expression of RmtB, RmtA, ArmA, and KamB resulted in significantly higher survival rates of 50.9%, 43.5%, 38.8%, and 30.6%, respectively. The remaining tested methyltransferases, RmtC, RmtD, Sgm, and NpmA, exhibited mixed effects on acid tolerance with diverse survival rates ranging from 8.4% to 24.9%.

## DISCUSSION

### Growth competition assay

Previous studies have investigated the fitness costs and physiological impacts of 16S rRNA methyltransferases that confer high-level aminoglycoside resistance, thereby offering valuable background for the present study. Lioy et al. observed pronounced fitness cost in cells expressing ArmA methyltransferase, whereas the expression of NpmA methyltransferase led to only slight fitness cost, causing decreased translational accuracy (33). Afterward, Ishizaki et al. illustrated that the expression of NpmA methyltransferase destabilizes bacterial genomes through the mobilization of insertion sequence elements, further compromising fitness in pathogenic *E. coli*, *P. aeruginosa*, and *K. pneumoniae* strains (36). Interestingly, some studies have shown that not every aminoglycoside resistance methyltransferase imposes a considerable impact on the level of bacterial fitness. Gutierrez et al. demonstrated that RmtC methyltransferase, which methylates nucleotide G1405 of the 16S rRNA, inhibits the modification of C1407 by RsmF and does this with less fitness costs to bacteria, even in the absence of the selective pressure of antibiotics. The authors observed a slight fitness cost associated with the expression of RmtC methyltransferase in both wild-type *E. coli* cells, as well as *E. coli* cells lacking the housekeeping methyltransferase RsmF (35). This contrasts with findings by Ou et al., who indicated that *E. coli* strains expressing the RmtB methyltransferase show considerable fitness costs (37). These dissimilar results suggest that specific structural or functional characteristics of different methyltransferases may determine their impact on bacterial fitness, highlighting the complex nature of the resistance-fitness trade-offs in clinical isolates.

Building on this foundation, our study provides a comprehensive analysis of eight 16S rRNA methyltransferases that confer high-level aminoglycoside resistance, and their implications on bacterial fitness. Our research interest was centered on two methyltransferases, Sgm and KamB, obtained from natural antibiotic producers, and six other enzymes (ArmA, RmtA, RmtB, RmtC, RmtD, and NpmA) isolated from clinical bacterial pathogens. Using an approach that included growth competition assays, translational fidelity measurements, and stress response assessments, our aim was to elucidate the specific nature and extent of the impact of these methyltransferases on bacterial fitness.

The results of growth competition assays revealed considerable variations in fitness costs among different methyltransferases. RmtA, RmtB, NpmA, and ArmA imposed the highest costs, effectively eliminating resistant strains from non-selective environments. Sgm and KamB showed milder fitness costs, allowing resistant strains to persist at detectable levels. RmtD was in an intermediate position since resistant populations underwent gradual reduction, albeit less severe than those expressing the costliest enzymes.

Comparison of our fitness cost study results with previous studies revealed that ArmA imposes a significant fitness burden, supporting the report of Lioy et al. (33). Similarly, RmtB showed a significant fitness cost, consistent with the report of Ou et al. (37). In contrast, our results indicate a significant fitness cost for RmtC; however, Gutierrez et al. described a lesser effect (35). The literature for NpmA is conflicting; our results, together with the report of Ishizaki et al., revealed a significant cost, while Lioy et al. reported a moderate effect (33, 36). These discrepancies, such as the reduced cost reported by Gutierrez et al., may arise from the differences in gene localization. Namely, the insertion of the methyltransferase gene into the chromosome specifically leads to lower and stable expression, thereby reducing the metabolic burden. This contrasts with the plasmid-encoded expression systems used in our study, which generally have high copy numbers, higher metabolic load, and numerous other plasmid-associated burdens. In addition, clinical isolates often harbor multiple resistance mechanisms and mutations that may heighten fitness costs compared to laboratory strains. These include the co-expression of other resistance genes on plasmids and hospital-acquired adaptive mutations, which may add costs to those observed in our controlled *E. coli* background.

In our experimental design, we employed the broad-host-range plasmid pBBR1MCS-4_START, in which the methyltransferase gene expression is controlled by a derepressed lac promoter. This derivative of the *E. coli* lac promoter functions in a near-constitutive manner due to the lack of the *lacI* repressor gene on the plasmid, resulting in insufficient repression by chromosomal *lacI* in *E. coli* hosts and allowing long-term high-level expression without an inducer like IPTG. This high-level expression may contribute to elevated fitness costs, a limitation we acknowledge as we lack direct measurements to confirm comparability across constructs.

It is interesting that, in clinical settings, genes for 16S rRNA methyltransferases are mostly found on plasmids (10, 17, 18, 40–45). This finding confirms the relevance of our experimental system, which utilizes plasmid-based expression as a suitable model for examining the fitness costs associated with these resistance determinants.

These results highlight the complex balance between the maintenance of antibiotic resistance and the resulting metabolic costs, thus providing valuable insight into the evolutionary processes governing resistance genes under non-selective conditions. The relatively low fitness costs of Sgm and KamB are probably reflective of co-evolutionary compromises by naturally occurring antibiotic-producing organisms to avoid interference with essential cellular processes. In contrast, the higher costs imposed by RmtA, RmtB, NpmA, ArmA, and RmtD suggest these more recently emerged resistance determinants have not yet developed mechanisms to minimize their metabolic costs. This evolutionary difference has significant implications for resistance stability in non-selective environments and may assist in explaining why certain methyltransferases are more commonly found in the clinic despite imposing a higher fitness cost.

### Translational fidelity

Translational fidelity assays were carried out using both *E. coli* DH5α strains and *E. coli* mutants lacking the gene encoding the endogenous 16S rRNA methyltransferase RsmF involved in methylation of nucleotide C1407 during the process of 30S ribosomal subunit assembly. Our observations revealed that even though the three-dimensional structures of these aminoglycoside resistance methyltransferases are similar, their impact on translational accuracy differs significantly. In the wild-type *E. coli* DH5α cells, most of the methyltransferases (Sgm, RmtA, RmtB, RmtC, ArmA, and NpmA) created error-prone phenotypes by encouraging read-through of nonsense and missense codons and causing frameshift errors. In contrast, KamB significantly enhanced translational fidelity, suggesting a specialized evolutionary adaptation in this natural producer-derived enzyme.

These varied effects can result from subtle differences in ribosomal binding sites and catalytic activities of the enzymes, highlighting an important evolutionary trade-off between the acquisition of aminoglycoside resistance and the maintenance of translational fidelity. The inherently error-prone phenotype seen in most methyltransferases suggests that these enzymes disrupt the complex proofreading mechanisms of the ribosome, perhaps by altering critical ribosomal structures or inhibiting important endogenous methylation at nearby nucleotides.

The differential effects seen within the *ΔrsmF* genetic background provide valuable information about the interactions between exogenously expressed resistance methyltransferases and endogenous rRNA modifications. The loss of the RsmF methyltransferase altered the error profiles caused by different resistance methyltransferases, thus highlighting the intricate interactions at the ribosomal level. For instance, the UGA readthrough caused by ArmA was reduced by 23% in the *ΔrsmF* strain, which indicates that the intrinsic methylation mediated by RsmF normally augments the error-inducing activity of ArmA. On the other hand, the effect of Sgm on CUG initiation was increased by 35%, whereas the effect of RmtC on −1 frameshift errors was increased by 40% in the *ΔrsmF* background, suggesting that the loss of C1407 methylation creates a more accommodating setting for some translational errors in the presence of these resistance methyltransferases.

The results reported in this work are in line with previous research regarding the interaction between housekeeping methyltransferases and aminoglycoside resistance methyltransferases. In previous studies, it was shown that Sgm methyltransferase suppresses the methylation activity of the housekeeping enzyme RsmF (34). Similarly, Gutierrez et al. showed that the RmtC methyltransferase also blocks the methylation of nucleotide C1407 by RsmF methyltransferase (35). More recently, Lioy et al. reported that ArmA methyltransferase specifically blocks the methylation activity carried out by RsmI at C1402, in contrast with previous speculations concerning C1407; moreover, NpmA directly blocks the activity of RsmF at the C1407 site (33). Taken together, these findings reveal the multiple mechanisms by which resistance methyltransferases interact and interfere with the normal processes of endogenous rRNA modifications.

The most notable result is the increased error-restrictive phenotype displayed by KamB in the *ΔrsmF* mutant, which saw a 40% decrease in frameshift errors compared to 22% seen in the wild-type strain. This result indicates that the unique ability of KamB to improve translational fidelity is further increased in the absence of RsmF-mediated methylation, which might be a result of compensatory processes or altered interactions with the ribosome. The new properties expressed by KamB, conferring resistance while at the same time improving translational accuracy, are an interesting adaptation worthy of further exploration.

The complex interplay between methyltransferase activity and ribosomal function, as explored here, provides critical insights into the molecular consequences of the acquisition of aminoglycoside resistance. Such findings increase our understanding of the trade-offs involved in resistance and fitness and identify likely vulnerabilities that could be exploited in the design of novel therapeutic strategies for the targeting of aminoglycoside-mediated resistance.

### Stress-response modulation

Results of the assays developed to measure the stress response revealed intriguing and complex effects of 16S rRNA methyltransferases on bacterial physiology under the challenge imposed by various environmental stressors. Our findings highlight the complex and varied roles that these enzymes play in bacterial adaptation, which extends beyond their primary function in conferring aminoglycoside resistance.

The results of this study revealed that most methyltransferases notably impaired the ability of *E. coli* to resist hyperosmotic stress to varying degrees. Strains expressing Sgm, RmtA, and KamB showed severely reduced growth (38.6–44.5% of wild-type levels) upon exposure to hyperosmotic stress. RmtB, RmtD, and ArmA exhibited moderate growth inhibition (53.6–62.0% of wild-type levels), while RmtC showed a milder effect (71.7% of wild-type growth). This observed susceptibility suggests that the methyltransferases can impair cellular processes that are related to osmoregulation. The high impact can be due to changes in the translation of osmoadaptation-related genes such as those coding for compatible solute transporters or enzymes that are involved in osmolyte biosynthesis (46). It was also recently shown that the hyperosmotic stress has an effect on bacterial protein synthesis by substantially reducing the translational elongation rate (47).

Notably, the strain expressing NpmA methyltransferase maintained growth rates similar to wild-type under hyperosmotic stress conditions (92.2% of wild-type growth). This difference in osmotic stress tolerance suggests that different methyltransferases might have different interaction sites or modes of action on the ribosome, having a divergent impact on cellular physiology. The ability of NpmA-expressing cells to maintain osmotic stress tolerance is of particular interest and warrants further investigation into possible compensatory mechanisms or special ribosomal interactions that maintain osmoregulatory functions.

In stark contrast to findings linked to hyperosmotic stress, the expression of several methyltransferases enhanced acid tolerance in *E. coli*. Strains carrying RmtB, RmtA, ArmA, and KamB methyltransferase showed high levels of survival (50.9%, 43.5%, 38.8%, and 30.6%, respectively) upon exposure to acid shock compared to wild-type *E. coli* cells (26.3%). The acid and hyperosmotic conditions simulated in our study mirror the environmental stresses and physiological demands found in the human gastrointestinal tract, as represented by the high acidity in the upper intestine and the high osmolarity in the colon. This suggests that increased acid tolerance may provide a selective advantage for survival and establishment of bacteria in the upper gastrointestinal tract, potentially impacting the spread of resistance in clinical settings. The increased resistance found at acidic pH values indicates that these methyltransferases may be enhancing the expression or activity of processes that counteract acid stress in *E. coli*. This observation is in agreement with the findings of a comprehensive analysis of transcriptomic and translational profiles in *E. coli* exposed to mild and severe levels of acid stress. The results of this study demonstrated not only known acid defense pathways but also uncovered a significant group of uncharacterized genes and pathways relevant to the regulation of both mild and severe acid stress (48).

Such contrasting responses to varying conditions of stress indicate that the 16S rRNA methyltransferases perform complex roles in the regulation of bacterial physiology, beyond their established function of aminoglycoside resistance. The observed differential responses to stress could be the result of modification of ribosomal function that influences the translation of stress-response genes or the regulators required for the adaptation of cells to environmental stress. The variable effects observed with RmtC, RmtD, Sgm, and NpmA methyltransferases in relation to acid tolerance highlight the complex nature of the interactions of these methyltransferases with the cellular processes involved in stress response. This variability suggests that each methyltransferase can have a unique “fingerprint” of physiological effects, possibly tied to their unique sites of action on the ribosome or different influences on the translation of stress-related proteins.

Our combined results reveal a complex relationship between methyltransferase function, translational fidelity, and the stress response. The level of osmotic tolerance is decreased by most of the methyltransferases to varying degrees, with the most notable effects observed for Sgm, RmtA, and KamB, while NpmA methyltransferase retains wild-type levels of tolerance. This trend is primarily associated with changes that are consistent with a marked decrease in translational fidelity, particularly evident in the increased rates of −1 frameshift and UGA read-through events. In contrast, NpmA methyltransferase maintains the stress tolerance of the wild-type strain alongside high translational fidelity, highlighting the importance of accurate translation processes for adaptation to stress.

Under acid stress conditions, strains expressing RmtB, RmtA, ArmA, and KamB methyltransferase show higher survival rates despite reduced osmotic tolerance and a moderate impairment of translational fidelity. This suggests that acid resistance mechanisms may involve specialized or compensatory pathways. Conversely, RmtC and RmtD show the lowest survival under acidic conditions, which is consistent with a severe reduction in translational fidelity and competitive fitness. This observation is consistent with previous research demonstrating that 16S rRNA methyltransferases isolated from clinical pathogens (ArmA, RmtB, RmtC, and RmtD) differ in their ribosomal A site binding patterns compared to Sgm methyltransferase isolated from a natural aminoglycoside producer (39).

Methyltransferases that show major disruptive effects on translation often simultaneously impair the response to certain stress as well as overall fitness. However, this relationship is not universal across all stress types, as evidenced by the enhanced acid tolerance observed in some methyltransferase-expressing strains. NpmA methyltransferase displays a unique pattern, with cells expressing this enzyme maintaining both translational accuracy and stress tolerance, suggesting that NpmA may have evolved to minimize fitness costs while conferring aminoglycoside resistance. This finding is consistent with previous research showing that 16S rRNA methyltransferases conferring high-level aminoglycoside resistance can bind and modify 30S ribosomal subunits at a late stage in their assembly, which may contribute to their varying effects on cellular physiology (49).

### Implications for antibiotic resistance management

Our findings have valuable implications for antibiotic stewardship and the design of new therapeutic strategies. The elevated fitness costs of most clinical methyltransferases align with the hypothesis that reduced selective pressure by more sensible use of aminoglycosides could lead to the reduction of resistance over time. Decreasing selective pressure by minimizing antibiotic usage and the removal of antibiotic contamination in environmental habitats, including wastewater, agricultural runoff, and veterinary use, can eventually cause the frequency of resistance to decline gradually over time, both in clinical and environmental situations (50). This supports antibiotic cycling policy in the clinical setting, where restriction phases of aminoglycosides can reduce the prevalence of high-cost resistance determinants. However, the relatively low fitness costs of methyltransferases from natural producers (Sgm and KamB) indicate that some resistance determinants will be preserved even when selection is eliminated, creating reservoirs of resistance genes that can rapidly spread when selection is re-imposed.

The differential stress responses that were observed in our work also uncover therapeutic liabilities that can be targeted. For instance, the osmotic hypersensitivity of the majority of the methyltransferase-expressing strains leads us to predict that combination regimens with osmotic challenge will differentially suppress resistant bacteria. Similarly, the enhanced acid resistance conferred by some methyltransferases can impact their capacity to survive within particular anatomical niches, that is, the stomach or urogenital tract, and thereby have significance for the therapy of corresponding infections. Furthermore, addressing weaknesses linked to high-cost resistance mechanisms provides considerable opportunities for upcoming therapeutic approaches. For example, enhancing the fitness costs of antibiotic resistance in the gut can facilitate biorestoration of susceptible populations, as suggested by Baquero et al. (51).

These measures of resistance control might be complicated by the potential of bacteria to evade the fitness cost by introducing secondary mutations, as shown in current research (48). Compensation can maintain bacterial fitness without re-establishing resistance and could conserve otherwise harmful resistance determinants in the population. Uncovering the mechanisms for this adaptation compensation, in terms of interaction with particular methyltransferases, will also be key to successfully counter aminoglycoside resistance.

## MATERIALS AND METHODS

### Bacterial strains, plasmids, and growth conditions

Genes encoding 16S rRNA methyltransferases were cloned into the plasmid pBBR1-MCS4_START (38). This broad-host-range plasmid, which is a derivative of the pBBR1MCS family, was selected for its stability and compatibility across gram-negative bacteria, including *E. coli*. Expression of cloned genes in the pBBR1MCS-4_START plasmid is driven by a derepressed *lac* promoter, a modified *E. coli lac* promoter that functions in a near-constitutive manner due to the absence of the *lacI* repressor gene on the plasmid. As a result, chromosomal *lacI* in *E. coli* cells provides inadequate repression, allowing continuous expression of downstream genes without the need for an inducer IPTG.

Plasmids pSG 3/4, pSG 413, and pSG 12DP used in translational fidelity assays were kindly provided by the late prof. Albert E. Dahlberg (Brown University, Providence, RI, USA) (52, 53).

The *sgm* and *npmA* genes were amplified from the constructs that were previously made in our laboratory (54, 55). Methyltransferase genes *armA*, *rmtB*, *rmtC,* and *rmtD* were kindly provided by Dr. Bruno Gonzalez-Zorn (Universidad Complutense de Madrid, Spain), while *rmtA* and *kamB* methyltransferase genes were kindly provided by Dr. Janusz M. Bujnicki (International Institute of Molecular and Cell Biology in Warsaw, Poland).

Methyltransferases were expressed in *E. coli* DH5α and *E. coli* BW25141 cells carrying the deletion of *rsmF* (56), and grown in Luria-Bertani (LB) Lennox broth or agar base (Invitrogen) with the addition of ampicillin (Sigma) (100 µg/mL). For translation accuracy experiments, *E. coli* cells expressing 16S rRNA methyltransferases and carrying either pSG 3/4, pSG 413, or pSG 12DP plasmids were grown in LB medium with the addition of both ampicillin (Sigma) (100 µg/mL) and tetracycline (10 µg/mL).

### Cloning of the methyltransferase genes

Methyltransferase genes were cloned into the pBBR1MCS-4_START plasmid, as described in our previous work (39). For cloning experiments, we used the restriction enzymes purchased from New England Biolabs. PCR products were amplified with the Phusion High Fidelity DNA Polymerase from Finnzymes. Plasmid constructs were confirmed by DNA sequencing (Macrogen, Korea).

### Growth competition assay

To determine the impact of 16S rRNA methyltransferases on the growth of bacteria, we employed a growth competition assay with modifications following the protocol of Gutgsell et al. (57). This procedure enables the quantitative measurement of growth defects or advantages caused by the expression of methyltransferases in competition with the control strains over several growth cycles. *E. coli* DH5α strains, either carrying the methyltransferase-expressing plasmids or empty-vector controls, were inoculated from single colonies into 3 mL of LB medium supplemented with ampicillin at 100 µg/mL and grown overnight at 37°C with shaking. Overnight cultures were adjusted to an optical density at 600 nm of 1.0 and underwent five consecutive 20-fold dilutions. 200 µL of the last dilution, containing approximately 3.2 × 10^6^ cells/mL, was plated on LB agar with ampicillin (100 µg/mL), to select for all plasmid-bearing cells, or on LB agar with both ampicillin (100 µg/mL) and kanamycin (50 µg/mL), to specifically select for strains expressing 16S rRNA methyltransferases. The plates were incubated at 37°C for 20 h, and the colonies were counted to establish baseline populations.

Equal volumes of control and methyltransferase-expressing cultures were mixed and diluted as described above. The mixture of cultures was diluted 1:1,000 in 20 mL LB medium and grown for 24 h at 37°C with shaking. The culture was then grown to OD_600_ of 1.0, diluted, and plated on selective medium as described above. The cycle was repeated 10 times to examine population dynamics over 10 generations. All growth competition experiments were repeated at least three times for each methyltransferase.

Following each growth cycle, the colony numbers were used to calculate the relative proportions of methyltransferase-producing strains to control strains. To calculate the competitive index (CI), we took the ratio of CFUs with methyltransferase expression divided by that of control strains and normalized the value to the initial ratio on Day 0. The selection coefficient (s) was derived from the slope of linear regression of ln(CI) against generation number, where negative values reflected the fitness cost of methyltransferase expression (29).

### Translational fidelity

To determine the effect of 16S rRNA methyltransferases on translational fidelity, we used a reporter system with modified *lacZ* alleles. Three reporter plasmids, pSG 3/4 carrying a UGA stop codon, pSG 413 carrying a CUG codon mutation, and pSG 12DP carrying a −1 frameshift mutation, were individually transformed into both *E. coli* DH5α and E. *coli* BW25141 *ΔrsmF* strains. The resulting strains were additionally transformed with pBBR1-MCS4_START plasmids carrying various methyltransferases (ArmA, RmtA, RmtB, RmtC, RmtD, NpmA, Sgm, and KamB) or empty-vector controls. The transformants were selected on LB agar plates supplemented with ampicillin (100 µg/mL) and tetracycline (10 µg/mL) to ensure the maintenance of both plasmids.

β-galactosidase assays were performed with a slight modification of the protocol of Miller et al. (58). Single colonies were inoculated into LB medium supplemented with ampicillin (100 µg/mL) and tetracycline (10 µg/mL) and grown overnight at 37°C with shaking. Cultures were diluted 1:100 in fresh medium and grown to mid-log phase (OD_600_ = 0.5–0.7). Cells were incubated on ice for 20 min to stop growth, and at least 2 mL of culture was centrifuged at 6,000 rpm for 10 min at 4°C. Cell pellets were resuspended in the same volume of chilled Z buffer (60 mM Na_2_HPO_4_, 40 mM NaH_2_PO_4_, 10 mM KCl, 1 mM MgSO_4_, and 50 mM β-mercaptoethanol, pH 7.0). The OD_600_ of the resuspended cells was measured against Z buffer as a blank. Cells were diluted to a final volume of 1 mL in Z buffer. Permeabilization of diluted cells was achieved by adding 100 µL chloroform and 50 µL 0.1% SDS, with vortexing. Samples were equilibrated for 5 min in a 28°C water bath.

The enzyme reaction was started by adding 0.2 mL of *o*-nitrophenyl-β-d-galactoside, ONPG (4 mg/mL in 0.1 M phosphate buffer, pH 7.0). The samples were incubated at 28°C until sufficient yellow color was produced (OD_420_ = 0.6–0.9). The reaction was terminated by the addition of 0.5 mL of 1 M Na_2_CO_3_. Following vortexing, 1 mL of each sample was placed in a microcentrifuge tube and centrifuged at top speed for 5 min to pellet cell debris and chloroform. The optical density was read at 420 and 550 nm for all samples, and Miller units were calculated accordingly (58). The assays were performed in three independent experiments.

### Stress-response assays

To assess the tolerance of bacteria to hyperosmotic stress, we adapted an osmotic shock assay protocol that was performed as follows (59). Overnight cultures of *E. coli* strains carrying various methyltransferases were diluted 1:100 in fresh LB medium and incubated until they reached the mid-logarithmic growth phase (OD_600_ = 0.5). Hyperosmotic stress was then induced by adding a 6 M solution of sodium chloride to achieve a final concentration of 300 mM in the culture. Bacteria were incubated at 37°C for 3 h with shaking at 200 rpm. Following the stress period, the samples were collected (*t* = 3 h) and serially diluted in sterile phosphate-buffered saline (PBS). Appropriate dilutions were plated on LB agar supplemented with the corresponding antibiotics to maintain plasmid selection. Colony-forming units (CFUs) were counted after overnight incubation at 37°C. Survival rates for each strain were normalized to the wild-type strain, whose post-stress viability was set to 100%. The assays were performed in three independent experiments.

Acid tolerance was determined using the adaptation of protocols described in previous studies (60, 61). Bacterial cultures were grown to mid-logarithmic phase (OD_600_ = 0.5) in LB medium at 37°C. To induce acid stress, 10 mL of culture was centrifuged at 6,500 g for 10 min. The supernatant was carefully removed, and the cell pellet was carefully resuspended in 10 mL of LB medium, and the pH was adjusted to 3.0 with HCl. Acid-challenged cultures were shaken at 200 rpm at 37°C for 1 hour. Afterward, the samples were collected, serially diluted in PBS, and plated on LB agar plates containing corresponding antibiotics to maintain plasmid selection. The number of viable cells was counted after overnight incubation at 37°C. Survival was calculated as a percentage of viable cells following acid challenge compared to cells at *t* = 0. The assays were performed in three independent experiments.

### Data visualization and statistical analysis

For statistical analysis and data visualization, we created custom Python scripts (Python 3.13.2, Python Software Foundation). Matplotlib (v3.10.1) (62) and Seaborn (v0.13.2) (63) libraries were used to create all figures, while NumPy (v2.2.3) (64), Statsmodels (v0.14.4) (65), and SciPy (v.1.15.2) (66) libraries were employed for data processing and statistical analysis. Statistical analyzes were performed to assess differences in growth and stress tolerance across strains. Two-way ANOVA was conducted to test for main effects and interactions between *E. coli* strain and plasmid type on relative growth rate, using the Statsmodels library (v0.14.4). Following ANOVA, post hoc Dunnett tests were applied to compare each methyltransferase-expressing strain against the control group, utilizing the scipy.stats module from the SciPy library (v1.15.2) with a two-sided alternative hypothesis. Error bars in figures represent the standard deviation of the mean, calculated using NumPy functions, to assess variability within replicates.

### Conclusion

Our results demonstrate that the acquisition of aminoglycoside resistance via 16S rRNA methyltransferases is marked by variable effects on fitness in *E. coli*. Results of growth competition assay established that each tested enzyme reduces bacterial fitness, with the most severe costs being imposed by methyltransferases isolated from the clinical setting (ArmA, RmtA, RmtB, RmtC, RmtD, and NpmA). In contrast, methyltransferases originating from environmental aminoglycoside-producing organisms (Sgm and KamB) caused relatively smaller decreases in fitness, allowing resistant populations to be maintained at reduced but stable levels in mixed culture. Experiments examining stress responses further revealed the complex nature of these compromises; while certain methyltransferases (RmtB, RmtA, ArmA, and KamB) increased survival during acidic stress conditions, they severely undermined tolerance to osmotic shock. Our results of translational accuracy assays disclosed that these enzymes have variable influences on ribosomal activity, with a majority (Sgm, RmtA, RmtB, RmtC, ArmA, and NpmA) causing erroneous phenotypes that contribute to elevated nonsense readthrough as well as frameshift mutations. Remarkably, KamB caused an opposite effect, supporting enhanced translational accuracy. Collectively, our results highlight the fragile balance between the advantages of resistance and the metabolic and physiological costs associated with the expression of methyltransferases. This research contributes to our knowledge of the molecular and evolutionary dynamics of aminoglycoside resistance and suggests that targeting special vulnerabilities associated with high-cost resistance mechanisms may provide valuable opportunities for therapeutic strategies against antibiotic-resistant infections.
